# Antimicrobial Activity of a Lipidated Temporin L Analogue against Carbapenemase-Producing *Klebsiella pneumoniae* Clinical Isolates

**DOI:** 10.3390/antibiotics10111312

**Published:** 2021-10-28

**Authors:** Emanuela Roscetto, Rosa Bellavita, Rossella Paolillo, Francesco Merlino, Nicola Molfetta, Paolo Grieco, Elisabetta Buommino, Maria Rosaria Catania

**Affiliations:** 1 Department of Molecular Medicine and Medical Biotechnology, University of Naples Federico II, Via S. Pansini 5, 80131 Naples, Italy; emanuela.roscetto@unina.it (E.R.); rossella.paolillo@unina.it (R.P.); 2 Department of Pharmacy, University of Naples Federico II, Via Montesano 49, 80131 Naples, Italy; rosa.bellavita@unina.it (R.B.); francesco.merlino@unina.it (F.M.); nicolamolfetta31@gmail.com (N.M.); paolo.grieco@unina.it (P.G.); elisabetta.buommino@unina.it (E.B.)

**Keywords:** *Klebsiella pneumoniae*, ESKAPE, multidrug resistance, carbapenemases, healthcare-associated infections, antimicrobial peptides, Temporin L

## Abstract

Over the years, the increasing acquisition of antibiotic resistance genes has led to the emergence of highly resistant bacterial strains and the loss of standard antibiotics’ efficacy, including β-lactam/β-lactamase inhibitor combinations and the last line carbapenems. *Klebsiella pneumoniae* is considered one of the major exponents of a group of multidrug-resistant ESKAPE pathogens responsible for serious healthcare-associated infections. In this study, we proved the antimicrobial activity of two analogues of Temporin L against twenty carbapenemase-producing *K. pneumoniae* clinical isolates. According to the antibiotic susceptibility assay, all the *K. pneumoniae* strains were resistant to at least one other class of antibiotics, in addition to beta-lactams. Peptides **1B** and **C** showed activity on all test strains, but the lipidated analogue **C** expressed the greater antimicrobial properties, with MIC values ranging from 6.25 to 25 µM. Furthermore, the peptide **C** showed bactericidal activity at MIC values. The results clearly highlight the great potential of antimicrobial peptides both as a new treatment option for difficult-to-treat infections and as a new strategy of drug-resistance control.

## 1. Introduction

In the order *Enterobacteriales*, *Klebsiella pneumoniae* is one of the most important causes of bloodstream, urinary and respiratory tract infections in vulnerable hosts [1]. An empirical antibiotic treatment is often required due to the severity of the infections and/or the patient’s critical conditions and the use of broad-spectrum antibiotics is necessary because of the possibility of a multidrug-resistant bacteria aetiology.

The worldwide spread of difficult-to-treat extended-spectrum beta-lactamase-producing enterobacteria has led to the use of carbapenems in empirical therapy [2], but the treatment with carbapenems has led to the rapid selection of carbapenem-resistant *Enterobacteriales* (CRE) [3].

*Enterobacteriales* may have different mechanisms of resistance to carbapenems. The most widespread one is the production of beta-lactamases with high affinity for carbapenems (carbapenemases). Another common mechanism of resistance is the hyperproduction of β-lactamases with limited affinity and/or hydrolytic activity toward carbapenems combined with structural alterations such as porin loss [4].

Carbapenemase production is also the most epidemiologically relevant resistance mechanism, as the genes for carbapenemases are carried by plasmids and therefore horizontally transmissible [5].

*Klebsiella pneumoniae* is the most common species harbouring transmissible carbapenemase [6].

In the Ambler classification system, carbapenemases are distributed in three classes depending on their chemical structure: classes A and D include serine-carbapenemases, whereas class B includes metallo-beta-lactamases [7]. *Klebsiella pneumoniae* carbapenemases (KPCs) are the most common transmissible genes among *Enterobacteriales* [8].

Treatment options for CRE are aminoglycosides, polymyxins or tigecycline, but some of these drugs have non-negligible adverse effects. Furthermore, many enterobacteria have additional plasmid-borne resistance genes, consequently resulting in resistance to several other antimicrobial groups. This issue induces clinicians to administer a combination therapy of two or more drugs [9]. The threat posed by CRE to human health is evidenced by their placement by WHO in the most critical group of multidrug-resistant bacteria for which the development of new antibiotics is urgently needed [10]. The new therapeutic options against CRE are drugs belonging to already known classes of antibiotics or new beta-lactam/beta-lactamase inhibitor combinations [11]. 

Antimicrobial peptides (AMPs) could provide a valid chance to overcome and control the antibiotic resistance [12]. Among these compounds, the temporins, isolated from the skin of *Rana temporaria* [13], represent one of the largest AMPs families. Temporin L (TL) is the most studied isoform for its potent activity both against Gram-positive and Gram-negative bacteria and yeasts. Due to its high cytotoxicity, TL has been the subject of different structure–activity relationship (SAR) studies to obtain novel analogues with an improved therapeutic index [14,15,16]. In this context, a previous SAR study consisting of the application of lipidation strategy on a potent Temporin L analogue, named peptide 1B [17,18], has led to the discovery of the lipidated peptide C featured by an alkyl chain of five carbons in para position of Phe^1^ in its N-terminus [19]. The addition of fatty acid conferred to peptide C self-assembling properties improved the effectiveness in inhibiting the growth of both *Staphylococcus aureus* (ATCC 25923) and *Klebsiella pneumoniae* (ATCC BAA-1705) cells, with a minimum inhibitory concentration (MIC) of 6.25 μM. Interestingly, it did not show a significant cytotoxic effect even at the high concentration of 25 μM [19]. In this study, we evaluated the activity of peptides 1B and C towards clinical carbapenem-resistant *K. pneumoniae* isolates harboring kpc or metallo-beta-lactamase genes.

## 2. Results

### 2.1. Antibiotic Susceptibility and RAPD Profiles

Twenty-three *K. pneumoniae* clinical strains were tested in this study and their sources are listed in Table 1. The KPC*Kp*1–KPC*Kp*18 and KN*Kp* strains all come from the Intensive Care Unit (ICU), whereas the strains VIM*Kp*, NDM*Kp*1, NDM*Kp*2 and NDM*Kp*3 come from different wards.

The conventional antibiotic susceptibility was tested for the following antibiotics: amoxicillin/clavulanic acid, cefotaxime, ceftazidime, piperacillin/tazobactam, gentamicin, amikacin, trimethoprim-sulfamethoxazole, ciprofloxacin, meropenem, ertapenem and ceftazidime/avibactam. The clinical strains were classified in different category S/I/R (susceptible/intermediate/resistant) according to EUCAST 2021 breakpoints (https://www.eucast.org/clinical_breakpoints, accessed on 28 April 2021). Table 2 shows the antimicrobial susceptibility profile of clinical strains: except for KN*Kp*, all the isolates were resistant to amoxicillin/clavulanic acid, ceftazidime, cefotaxime, piperacillin/tazobactam and to the carbapenems ertapenem and meropenem (except for KPC*Kp*6 and KPC*Kp*10 that showed an intermediate resistance to meropenem). They were also resistant to ciprofloxacin, excluding KPC*Kp*5 and NDM*Kp*2. Particularly, KPC*Kp*8 and KPC*Kp*15 showed a resistant profile towards all tested antibiotics. We observed that the aminoglycosides amikacin and gentamicin and the combination ceftazidime/avibactam were the most effective compounds, acting against 52.2% (12/23), 56.5% (13/23), 8.7 % (2/23) of clinical strains, respectively. The 22 carbapenem-resistant strains were subjected to molecular tests (Xpert Carba-R-test), which allow the identification of the resistance determinants involved (KPC, VIM, IPM-1, NDM, OXA-48). We found that 81.8% of the strains (named KPC*Kp*1–KPC*Kp*18) produced KPC-type carbapenemases, whereas 18.2% produced VIM (VIM*Kp*) or NDM (NDM*Kp*1, NDM*Kp*2, NDM*Kp*3) metallo-beta-lactamases (MBL). Overall, 99% of the *K. pneumoniae* clinical isolates were multidrug-resistant (MDR, resistant to at least three antibiotics belonging to different antibiotics categories). 

**Figure 1:**
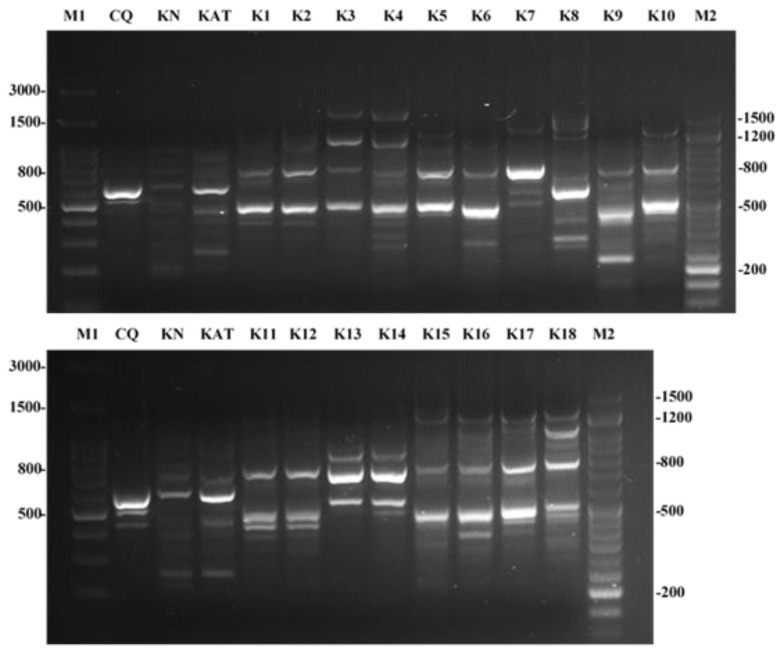
RAPD analysis of both ATCC and clinical strains from ICU. M1: 100 bp ladder; M2: 50 bp.

All the *K. pneumoniae* clinical strains coming from the same ward (ICU) (KPC*Kp*1–KPC*Kp*18) were genotyped through the Random Amplified Polymorphic DNA (RAPD) analysis based on the Polymerase Chain Reaction technique (PCR), and the genomic profiles were compared with the antibiotic susceptibility patterns. As shown in Figure 1, the strains showed intra-specific variations between the genomic profiles, except for KPC*Kp*1 and KPC*Kp*2, sharing the same antibiotic susceptibility profile (Table 2), as well as KPC*Kp*13 and KPC*Kp*14. KPC*Kp*5 showed a genetic profile comparable to that of KPC*Kp*1 and KPC*Kp*2, but a different susceptibility to GM, SXT and CIP. Similarly, KPC*Kp*11 and KPC*Kp*12 showed a comparable genetic profile, but differences in sensitivity to GM and SXT. Regarding the strains KPC*Kp*15 and KPC*Kp*16, RAPD analysis showed some similarity, but by comparing their antibiotic susceptibility profile we found that these were different. KPC*Kp*15 showed resistance to GM, AK and CAZ/AVI, whereas KPC*Kp*16 did not. On the contrary, KPC*Kp*17 and KPC*Kp*18 showed a similar profile for antibiotic susceptibility, but a different RAPD profile. Thus, from the results of RAPD analysis, we chose to work only on 21 KPC*Kp* strains that showed both different genetic and antibiotic susceptibility profiles, excluding the strains KPC*Kp*2 and KPC*Kp*14. The strains selected were further investigated.

### 2.2. Antimicrobial Activity of Peptides

The antimicrobial activity of peptides **1B** and **C** was tested against ATCC 13883 (KCQ) and ATCC BAA-1705 (KAT) as *K. pneumoniae* reference strains and *K. pneumoniae* clinical isolates. The peptides resulted to be active against all the tested strains and MIC values are reported in Table 3. Both peptides inhibited the growth of carbapenem-sensitive strains (KCQ and KN*Kp*) at MIC values of 6.25 µM; on the other hand, both peptides were able to effectively inhibit the growth of carbapenemase-producing strains with MIC values ranging from 12.5 µM to 100 µM for peptide **1B**, and MIC values ranging from 6.25 µM to 25 µM for peptide **C**. Furthermore, peptide **C** showed bactericidal activity at MIC values, whereas **1B** was bacteriostatic at MIC values, and bactericidal at 2 × MIC values.

## 3. Discussion

*Klebsiella pneumoniae* is a frequent colonizer of the human gut and a major cause of healthcare-related infections whose treatment is complicated by the constant increase in antibiotic resistance. *K. pneumoniae* was included in the “ESKAPE” group (*Enterococcus faecium*, *Staphylococcus aureus*, *K. pneumoniae*, *Acinetobacter* *baumannii*, *Pseudomonas aeruginosa* and *Enterobacter species*) [18]. These pathogens acquired resistances through time, becoming one of the major health concerns of the modern day.

Carbapenems represent the last-resort beta-lactams, and carbapenem-resistant *K. pneumoniae* strains are currently spread all over the world [8]. The most relevant mechanism of carbapenem resistance is the production of carbapenemases. *Klebsiella pneumoniae* carbapenemases (KPCs) are the most common enzymes reported worldwide and capable of deactivating all of the beta-lactams [20]. Among the metallo-beta-lactamases (MBL), New Delhi MBL (NDM), Verona integron-encoded MBL (VIM) and imipenemase MBL (IMP) are the most common enzymes identified worldwide [8]. MBL-producers are continuously isolated in new regions, notably *K. pneumoniae* strains harboring the *ndm* gene [21].

The worrying spread of carbapenemase-producing enterobacteria is due to the prevalent localization of these genes on mobile genetic elements. *K. pneumoniae* can both acquire and carry a great number of genetic mobile elements, thus accumulating resistance genes and expanding its accessory genome, with the evolution of multi drug- and extensively drug-resistant strains [22].

Most of the recently approved drugs for the treatment of CRE are new combinations of an old beta-lactam with a second-generation beta-lactam inhibitor (BL/BLI). These combinations are ineffective on MBL-producing strains, whereas they generally show activity on strains harboring KPC carbapenemases [23]. Among these new combinations, ceftazidime/avibactam was approved by the FDA in 2015. Although it was recently introduced, KPC-producing *K. pneumoniae* isolates resistant to ceftazidime/avibactam have already been reported [24,25].

For that reason, the attention shifted on the identification of natural-derived peptides, whose mechanisms of action strongly differ from the classic antibiotics.

Among the novel generations of antimicrobial compounds, the antimicrobial peptides (AMPs) play a significant role in this context [26,27]. AMPs are widely produced by different kinds of living forms, and their structure typically consist of a variable-length amino acids chain (10 to 60 a.a.) [28,29]. The positive charge due to the presence of basic residues (lys and arg), the hydrophobic residues (about 50%) and the amphipathic nature are commonly shared features that characterize those molecules [30]. Considering that the bacterial membrane has been identified as a physical target of AMPs [31], the development of resistance mechanisms is greatly hampered. On the other hand, the cellular toxicity and the pharmacokinetic issues represent the main drawbacks of these compounds [32]. To augment their activity against bacteria and decrease the cytotoxicity, lipidation strategy was employed [19]. 

In our previous study, two derivatives of the Temporin L from *Rana temporaria*, named **1B** and **C**, were tested on *Pseudomonas aeruginosa*, *Klebsiella pneumoniae* and *Staphylococcus aureus*, showing good activity on these pathogens. Based on these preliminary results, in this study the peptides **1B** and **C** were evaluated on 20 clinical strains of *K. pneumoniae,* all carbapenemase-producers. Of these, 16 strains carried a KPC carbapenemase, whereas four isolates harbored an MBL. Initially, 18 KPC-producing clinical strains were included in the study, all from the intensive care unit. They were genotyped by RAPD: most of the tested strains showed different RAPD profiles, confirming the high heterogeneity of *K. pneumoniae* [33]. For those strains showing comparable RAPD profiles, antibiotic-susceptibility patterns were considered. On this basis, strains 2 and 14 were excluded as both RAPD and antibiotic-susceptibility profiles were comparable to strains 1 and 13, respectively. *K. pneumoniae* strains with comparable RAPD profile, but with different antibiotic-susceptibility pattern for at least one interpretative category were instead included in the study.

Peptides **1B** and **C** showed activity on all the tested strains. The lipidated analogue **C** was more active than peptide **1B**, probably due to the modification applied on its molecular structure, with MIC values ranging from 6.25 to 25 µM against the KPC-producing strains, and MIC values of 25 µM against the MBL-producing strains. Interestingly, at the highest concentration of 25 µM used in this study, it has previously been shown that peptide **C** was not cytotoxic both on human keratinocytes and erythrocytes [19]. MBL producers are very difficult to treat as therapeutic options are even more limited, but isolation of KPC-producing strains resistant to the new BL/BLI combinations complicates antibiotic treatment. In our study, 2 (KPC*Kp*8 and KPC*Kp*15) of the 16 KPC-producers were resistant to ceftazidime/avibactam. Moreover, these results seem particularly interesting to us as all the carbapenemase-producing strains tested were also resistant to classes of antibiotics other than beta-lactams. Notably, KPC*Kp*1 strain was also resistant to polymyxin E, drug shelved for its side effects and then reintroduced into human therapy as a salvage treatment against multidrug-resistant Gram-negative bacteria [34]; the KPC*Kp*15 strain was resistant to all drugs used.

## 4. Materials and Methods 

### 4.1. Synthesis

The peptides **1B** [H-Phe-Val-Pro-Trp-Phe-Ser-Lys-Phe-DLeu-DLys-Arg-Ile-Leu-NH_2_] and **C** [H-Phe(4-NHCO(CH_2_)_3_CH_3_)-Val-Pro-Trp-Phe-Ser-Lys-Phe-DLeu-DLys-Arg-Ile-Leu-NH_2_] were synthesized using Fmoc-based ultrasonic-assisted solid phase peptide synthesis (US−SPPS) methodology [35]. The elongation of the peptide sequence consisted in repeated cycles of Fmoc-deprotection and coupling reactions. Specifically, the Fmoc group was removed treating the resin with a solution of 20% piperidine in DMF (0.5 × 1 min) by ultrasonic irradiations, whereas each coupling reaction was performed using N^α^-Fmoc-amino acid (3 equiv), HBTU (3 equiv), HOBt (3 equiv) and DIEA (6 equiv) in DMF for 5 min by ultrasound waves. After the peptide assembly, the conjugation of valeric acid in para position of Phe^1^ of peptide C was performed as previously reported [19]. In particular, the nitro group in para position of Phe^1^ was reduced treating the resin with a 1M solution of SnCl_2_ in DMF for 12 h and then, the valeric acid (3 equiv) was added using HBTU (3 equiv), HOBt (3 equiv) and DIEA (6 equiv) in DMF for 2 h on automated shaker. Finally, peptides were treated with a cleavage cocktail (TFA:TIS:H_2_O, 95:2.5:2.5) to be released from the resin and cleaved from their protecting groups, and then they were purified and characterized by RP-HPLC using linear gradients of MeCN (0.1% TFA) in water (0.1% TFA), from 10 to 90% over 20 min.

### 4.2. Bacterial Strains and Culture Conditions

Strains of *Klebsiella pneumoniae* evaluated in this study included reference strains such as carbapeneme-susceptible ATCC 13883 (KCQ) and carbapeneme-resistant ATCC BAA-1705 (KAT), and 23 clinical strains (Table 1) belonging to a collection of anonymous isolates, previously established at the Department of Molecular Medicine and Medical Biotechnology (University of Naples Federico II) during two-year period (March 2020–March 2021). Among the clinical strains, the first 19 listed strains all come from the Intensive Care Unit and all but one (KN*Kp*) were resistant to carbapenems. The last 4 listed strains were from different wards (Cystic Fibrosis Center, Oncology, Geriatrics, Cardiac Surgery) and were metallo-β-lactamases (MBLs) producers. Identification was performed by biochemical characterization using the Vitek II system (BioMérieux, Marcy-l’Étoile, France) and was confirmed by MS MALDI-TOF (Bruker Daltonics, Bremen, Germany). Antibiotic susceptibility profiles were assessed using automated systems (Vitek 2; Phoenix—Becton Dickinson, Sparks, NV, USA) and broth microdilution method. Carbapenemase gene detection was performed using the Xpert Carba-R-test (Cepheid, Sunnyvale, CA, USA), real-time PCR diagnostic tests that allow to detect and differentiate the most prevalent carbapenemase gene families. All strains were stored as 15% (*v*/*v*) glycerol stocks at −80 °C. Before each experiment, cells were sub-cultured from stocks on Tryptic Soy Agar (TSA) (Becton Dickinson) plates to 37 °C for 24 h.

### 4.3. Antimicrobial Assays: Determination of the Minimum Inhibitory Concentration (MIC) and the Minimum Bactericidal Concentration (MBC)

The antibacterial activity of peptides **1B** and **C** was determined using a standard method of microdilution in broth, following the procedure already described [36]. For each strain, the bacterial suspension was prepared at 0.5 McFarland standard (corresponding to about 10^8^ CFU/mL) in Mueller Hinton broth (MHB—Becton Dickinson) and subsequently adjusted to about 1.5 × 10^6^ CFU/mL. One hundred microliter aliquots of this suspension were dispensed into 96-well microtiter plates. A 2x stock solution of temporin was serially diluted (twofold dilutions) with MHB and added to the wells to a final concentration between 3.125 µM and 100 µM. The plates were incubated at 37 °C for 19 h with stirring (300 rpm). The turbidity of the medium was measured with a spectrophotometer at 595 nm (Bio-Rad Laboratories S.r.l., Hercules, CA, USA). Wells with only MHB were used as a negative control and wells without peptide as a growth control. Polymyxin E (Sigma-Aldrich, Milan, Italy) was selected as a control from the conventional antimicrobials and tested at concentrations ranging from 2 µg/mL to 8 µg/mL. The MIC was defined as the lowest concentration of the compound that resulted in 100% growth inhibition after 19 h of incubation. The MBC was determined by transferring 50 µL aliquots of each well with concentrations equal to or higher than the MIC, onto TSA plates and incubating the plates at 37 °C for 24 h. The lowest compound concentration that yielded no bacterial growth on agar plates was defined as the MBC. Each compound was tested alone in triplicate; each experiment was performed twice.

### 4.4. RAPD Analysis

Random Amplification of Polymorphic DNA was performed on both ATCC strains and clinical isolates. Among all the tested primer, the HI-RP (5′-AACTCGGCGACCAGCTACAA-3′) primer was selected and used for the amplification [15]. The final RAPD conditions for HI-RP were: 0.5 µL of genomic DNA, 20 µL H_2_O, 2.5 µL buffer, 1 μL dNTP, 1 µL primers and 0.1 µL of Taq DNA polymerase (Biotech Rabbit) in a final volume of 25 μL. The amplification program included an initial step at 94 °C for 5 min, followed by 40 cycles of 30 s at 95 °C, 1 min at 36 °C, and1 min at 72 °C, with a final extension cycle at 72 °C for 7 min. Reactions were performed using a thermo cycler (T100 THERMAL CYCLER-BioRad, Hercules, CA, USA). The PCR products were analysed by electrophoresis on 2% agarose gel in TBE and stained with ethidium bromide. The gels were photographed under UV light to record the results.

## 5. Conclusions

In conclusion, the obtained data show that the selected lipidated peptide C seems very promising for the development of a new drug with extensive antimicrobial activity and confirm and underline the potential role of temporins, and AMPs globally and efficiently counter the outbreaks of new multidrug-resistant pathogens. These compounds represent a valid mean to support both the management of serious infections and contrast the further expansion of antibiotic resistances.

## Figures and Tables

**Table 1:** *K. pneumonaie* clinical strains used in antimicrobial assays.

Strain Name	Source	Strain Name	Source
KN*Kp*	AF	KPC*Kp*12	TR
KPC*Kp*1	TR	KPC*Kp*13	E
KPC*Kp*2	TFr	KPC*Kp*14	B
KPC*Kp*3	UC	KPC*Kp*15	TF
KPC*Kp*4	UC	KPC*Kp*16	TR
KPC*Kp*5	AF	KPC*Kp*17	U
KPC*Kp*6	U	KPC*Kp*18	U
KPC*Kp*7	E		
KPC*Kp*8	TF	VIM*Kp*	AF
KPC*Kp*9	C	NDM*Kp*1	Tfr
KPC*Kp*10	E	NDM*Kp*2	U
KPC*Kp*11	P	NDM*Kp*3	E

Abbreviations: AF, pharyngeal aspirate; B, bronchus aspirate; C, catheter; CF, cystic fibrosis; E, blood colture; P, prothesis; TF, pharyngeal swab; TFr, wound swab; TR, rectal swab; UC, catheter urine; U, urine.

**Table 2:** Antibiotic-susceptibility profile of *K. pneumoniae* clinical strains.

Strains	AMC	CTX	CAZ	TZP	GM	AK	SXT	CIP	MEM	ERT	CAZ/AVI
KN*Kp*	4 (S)	≤1 (S)	≤0.5 (S)	≤4 (S)	≤1 (S)	≤4 (S)	≤1 (S)	≤0.25 (S)	≤0.125 (S)	≤0.25 (S)	1 (S)
KPC*Kp*1	>16 (R)	>32 (R)	>32 (R)	>64 (R)	≤1 (S)	≤1 (S)	4 (I)	>2 (R)	>8 (R)	>4 (R)	8 (S)
KPC*Kp*2	>16 (R)	>32 (R)	>32 (R)	>64 (R)	≤1 (S)	≤1 (S)	4 (I)	>2 (R)	>8 (R)	>4 (R)	1 (S)
KPC*Kp*3	>32 (R)	>4 (R)	>8 (R)	>16 (R)	≤1 (S)	4 (S)	>4 (R)	>1 (R)	>8 (R)	>1 (R)	4 (S)
KPC*Kp*4	>32 (R)	>4 (R)	>8 (R)	>16 (R)	>4 (R)	4 (S)	>4 (R)	>1 (R)	>8 (R)	>1 (R)	1 (S)
KPC*Kp*5	>16 (R)	>32 (R)	>32 (R)	>64 (R)	>8 (R)	4 (S)	>8 (R)	0.25 (S)	>8 (R)	>4 (R)	4 (S)
KPC*Kp*6	>32 (R)	>4 (R)	>32 (R)	>64 (R)	2 (S)	8 (S)	>4 (R)	>1 (R)	8 (I)	>1 (R)	1 (S)
KPC*Kp*7	>32 (R)	>4 (R)	>8 (R)	>16 (R)	4 (I)	≤4 (S)	≤1 (S)	>1 (R)	>8 (R)	>1 (R)	1 (S)
KPC*Kp*8	>16 (R)	>32 (R)	>32 (R)	>64 (R)	>8 (R)	32 (R)	>8 (R)	>2 (R)	>8 (R)	>4 (R)	>8 (R)
KPC*Kp*9	>16 (R)	>32 (R)	>32 (R)	>64 (R)	≤1 (S)	4 (S)	>8 (R)	>2 (R)	>8 (R)	>4 (R)	4 (S)
KPC*Kp*10	>16 (R)	>32 (R)	>32 (R)	>64 (R)	≤1 (S)	≤1 (S)	>8 (R)	>2 (R)	8 (I)	>4 (R)	4 (S)
KPC*Kp*11	>32 (R)	>4 (R)	>8 (R)	>16 (R)	>4 (R)	>16 (R)	>8 (R)	>1 (R)	>8 (R)	>1 (R)	1 (S)
KPC*Kp*12	>16 (R)	>32 (R)	>32 (R)	>64 (R)	2 (S)	>32 (R)	≤1 (S)	>2 (R)	>8 (R)	>4 (R)	4 (S)
KPC*Kp*13	>16 (R)	>32 (R)	>32 (R)	>64 (R)	>8 (R)	>16 (R)	>8 (R)	>2 (R)	>8 (R)	>4 (R)	4 (S)
KPC*Kp*14	>16 (R)	>32 (R)	>16 (R)	>64 (R)	>8 (R)	32 (R)	>8 (R)	>2 (R)	>8 (R)	>4 (R)	1 (S)
KPC*Kp*15	>16 (R)	>32 (R)	>32 (R)	>64 (R)	>8 (R)	32 (R)	>8 (R)	>2 (R)	>8 (R)	>1 (R)	>8 (R)
KPC*Kp*16	>16 (R)	>32 (R)	>32 (R)	>64 (R)	2 (S)	4 (S)	>8 (R)	>2 (R)	>8 (R)	>4 (R)	4 (S)
KPC*Kp*17	>16 (R)	>32 (R)	>32 (R)	>64 (R)	≤1 (S)	≤1 (S)	≤1 (S)	>2 (R)	>8 (R)	>4 (R)	4 (S)
KPC*Kp*18	>32 (R)	>4 (R)	>8 (R)	>16 (R)	2 (S)	4 (S)	≤1 (S)	>1 (R)	>8 (R)	>1 (R)	4 (S)
VIM*Kp*	>32 (R)	>4 (R)	>8 (R)	>16 (R)	2 (S)	≤4 (I)	>4 (R)	1 (R)	8 (R)	>1 (R)	>8 (R)
NDM*Kp*1	>16 (R)	>32 (R)	>32 (R)	>64 (R)	>8 (R)	16 (I)	≤1 (S)	>2 (R)	8 (R)	>1 (R)	>8 (R)
NDM*Kp*2	>16 (R)	32 (R)	>32 (R)	>64 (R)	>8 (R)	>16 (R)	>8 (R)	≤0.06 (S)	>8 (R)	>4 (R)	>8 (R)
NDM*Kp*3	>16 (R)	>32 (R)	>32 (R)	>64 (R)	>8 (R)	32 (R)	≤1 (S)	>2 (R)	>8 (R)	>1 (R)	>8 (R)

Abbreviations: AMC, Amoxicillin/clavulanic acid; AK, Amikacin; CAZ, Ceftazidime; CAZ/AVI, Ceftazidime/avibactam; CIP, Ciprofloxacin; CTX, Cefotaxime; ERT, Ertapenem; GM, Gentamicin; I, Intermediate; MEM, Meropenem; R, Resistant; S, Susceptible; SXT, Trimethoprim-Sulfamethoxazole; TZP, Piperacillin/tazobactam.

**Table 3:** Minimum inhibitory concentrations (MIC) of peptides **1B** and **C** against *K. pneumoniae* test strains.

Strains	MIC (µM)		MIC (µg/mL)
	1B	C	Polymyxin E
KCQ	6.25	6.25	<2 (S)
KAT	12.5	6.25	<2 (S)
KN*Kp*	6.25	6.25	<2 (S)
KPC*Kp*1	50	6.25	8 (R)
KPC*Kp*3	100	25	<2 (S)
KPC*Kp*4	25	25	<2 (S)
KPC*Kp*5	50	12.5	<2 (S)
KPC*Kp*6	12.5	12.5	<2 (S)
KPC*Kp*7	12.5	12.5	<2 (S)
KPC*Kp*8	50	12.5	<2 (S)
KPC*Kp*9	12.5	12.5	<2 (S)
KPC*Kp*10	50	12.5	<2 (S)
KPC*Kp*11	25	12.5	<2 (S)
KPC*Kp*12	25	25	<2 (S)
KPC*Kp*13	25	12.5	<2 (S)
KPC*Kp*15	50	25	8 (R)
KPC*Kp*16	25	25	<2 (S)
KPC*Kp*17	25	12.5	<2 (S)
KPC*Kp*18	25	12.5	<2 (S)
VIM*Kp*	50	25	<2 (S)
NDM*Kp*1	25	25	<2 (S)
NDM*Kp*2	50	25	<2 (S)
NDM*Kp*3	100	25	<2 (S)

Polymyxin E used as control conventional antimicrobial.

## Data Availability

Data are contained within the manuscript.
